# Métastase splénique d'un adénocarcinome colique - à propos d'un cas et revue de la littérature

**Published:** 2011-11-23

**Authors:** Fatima Zahra El M'rabet, Sami Aziz Brahmi, Siham Rachidi, Siham Tizniti, Afaf Amaarti, Khalid Ait Taleb, Omar El Mesbahi

**Affiliations:** 1 Service d'Oncologie Médicale, CHU Hassan II, Fès, Maroc; 2 Service de Radiologie, CHU Hassan II, Fès, Maroc; 3 Service d'Anatomo-pathologie, CHU Hassan II, Fès, Maroc; 4 Service de Chirurgie Viscérale B, CHU Hassan II, Fès, Maroc

**Keywords:** Adénocarcinome, rate, colon, métastase, cancer

## Abstract

L'atteinte métastatique de la rate est rare et exceptionnellement isolée. En effet, elle survient généralement dans le cadre d'une atteinte multi viscérale. Les cancers les plus pourvoyeurs de métastase splénique sont les mélanomes, les carcinomes de l'ovaire, du sein et du poumon. Dans le cancer colique, l'atteinte métastatique isolée de la rate est rare, dix cas seulement ont été décrits dans la littérature jusque-là. À travers cette revue, nous décrivons un nouveau cas présentant un adénocarcinome colique avec métastase splénique métachrone, tout en discutant les aspects cliniques et les différentes approches thérapeutiques décrites dans la littérature. Nous rapportons un nouveau cas d'un patient âgé de 46ans ayant un adénocarcinome colique traité, et qui a présenté 5 ans plus tard une métastase splénique de découverte fortuite lors d'un bilan radiologique de surveillance, pour laquelle le patient a bénéficié d'une splénectomie suivie d'une chimiothérapie systémique avec une bonne évolution. Les métastases spléniques isolées des tumeurs solides sont rares, et leur diagnostic est souvent de découverte fortuite. La splénectomie totale est un moyen efficace de faire le diagnostic définitif de ces métastases et de les traiter afin de prévenir les complications et d'améliorer la survie.

## Introduction

Les métastases spléniques isolées des tumeurs solides sont rares, et leur diagnostic est souvent de découverte fortuite. Celles du cancer colorectal sont exceptionnelles et surviennent généralement dans le cadre de l’atteinte disséminée. Bien que leur incidence exacte est encore inconnue, Berge a rapporté une incidence globale de 7,1% tout cancer confondu pour 7165 autopsies et l’incidence de métastase splénique de cancer colique est de 4,4% [1]. La survie après splénectomie chez les patients ayant une métastase splénique isolée d’un cancer colorectal est inconnu, néanmoins les données limitées de cas rapportés dans la littérature indiquent que ces patients peuvent survivre jusqu’à 7 ans [2].

## Patient et observation

Les auteurs déclarent avoir reçu le consentement écrit du patient pour reporter ce cas. Mr E.A âgé de 46 ans, alcoolo-tabagique chronique pendant 18 ans sevrés il y a 10 ans. Suivi depuis 2002 pour adénocarcinome lieberkuhnien moyennement différencié du colon droit. Il a bénéficié en 2002 d’une hémi colectomie droite. La tumeur a été classée T4 N0 M0 R0 (stade III). L’ACE (Antigène carcinoembroyonaire) était normal à 4 ng/ml. Le patient a reçu 6 cures de chimiothérapie adjuvante à base de 5 fluoro-uracile et d’acide folinique type FUFOL Mayo clinique.

**Figure 1:**
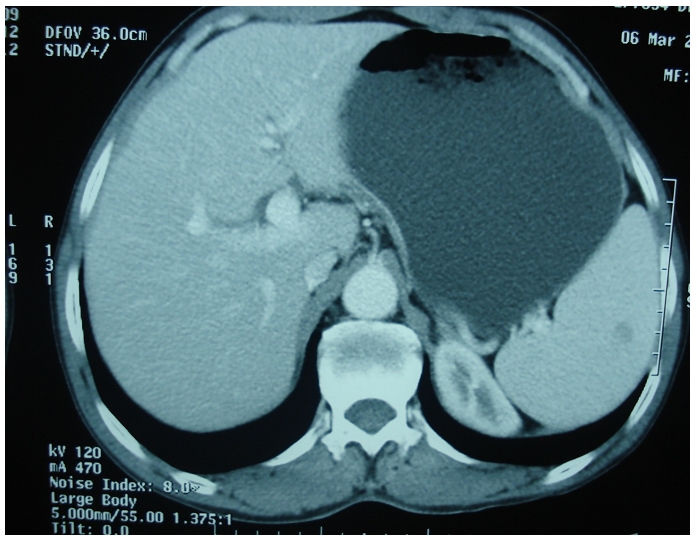
Coupe scannographique passant par la rate montrant un nodule splénique solitaire de découverte fortuite

**Figure 2:**
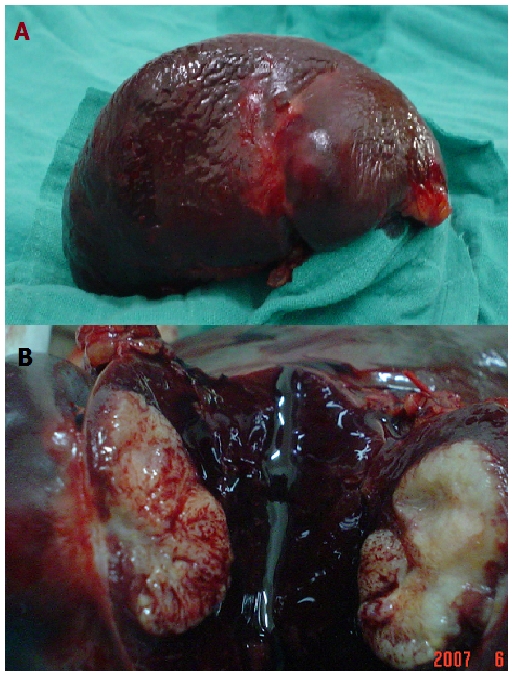
Aspect macroscopique du nodule splénique sur la pièce de splénectomie (A). Capsule intact après dissection de la pièce (B)

Une récidive au niveau de l’angle colique droit a été diagnostiquée 3 mois après la dernière cure de chimiothérapie avec ascension de l’ACE à 152 ng/ml, d’où l’indication d’une résection chirurgicale en 2003, suivie de 4 cures à base d’irinotecan (compto=350mg/m2, J1=J21) avec bonne réponse clinique et biologique. Après deux ans de rémission complète le patient a présenté des métastases hépatiques et costales révélées par des douleurs abdominales et objectivées par le scanner abdominal, pour lesquelles il a eu une résection chirurgicale en 2005: résection tumorale emportant une pastille du foie, le pôle supérieure du rein, la 11^e^ et la 12^e^ côte, la parois musculaire dont l’examen anatomopathologique a comfirmé la récidive, suivie de 6 cures de chimiothérapie à base de capécitabine (Xéloda 1250 mg X2/j). Une année après la dernière cure de capécitabine, au cours de la surveillance radiologique, un scanner thoraco-abdomino-pelvien fait en 2007 a objectivé un nodule splénique hypo dense de 11 mm, isolé (Figure 1), le complément échographique: un nodule splénique de 16 mm de diamètre présentant un aspect en cocarde avec un centre hyperéchogène et un halo périphérique hypoéchogène suspectant en premier une métastase splénique solitaire sans récidive colique ni autre atteinte métastatique. L’ACE était normal. Le patient a bénéficié d’une splénectomie en avril 2007: à l’exploration: la rate était siège de deux nodules d’allure secondaire (Figure 2), le reste de la cavité abdominale était sans particularité. L’examen anatomopathologique était en faveur de métastases spléniques d’un processus peu différencié glandulaire de 2,5 et 2,8 cm de grand axe compatible avec une origine colique. Le patient a reçu ensuite 8 cures de chimiothérapie adjuvante à base de capécitabine et d’oxaliplatine (XELOX/ Xéloda 1000mg/m2X2/j + Oxaliplatine: 130 mg/m2, J1=J21) avec une bonne tolérance clinique et biologique. Actuellement le patient est en rémission complète avec un recul de 3 ans.

## Discussion

Les localisations spléniques des maladies hématologiques malignes sont fréquentes. En revanche, celles des carcinomes sont beaucoup plus rares, elles ont une fréquence de survenue d’environ 7% tout cancer confondu [1]. Elles sont observées dans, les cancers du sein, l’ovaire, l’estomac, les mélanomes et surtout le poumon qui constitue le cancer primitif le plus fréquemment responsable de ces métastases [3]. L’incidence de métastase splénique de cancer colique est de 4,4% selon des séries d’autopsies [1]. Différentes théories expliquent cette rareté: d’une part l’existence de mécanismes inhibant la prolifération au niveau de la rate, et d’autre part l’absence de lymphatiques afférents à la rate. L’envahissement métastatique de la rate se fait préférentiellement par voie artérielle systémique soit après passage hépatique et pulmonaire ou soit, plus rarement, par l’artère splénique en court-circuitant le réseau hépato-pulmonaire. Cependant, la voie lymphatique rétrograde est, elle aussi, possible. Le faible taux de métastase splénique au regard de l’importante vascularisation splénique n’a pas, à ce jour, d’explication reconnue [4]. Les métastases spléniques surviennent, en général, à un stade évolué de la maladie cancéreuse, associées à d’autres atteintes métastatiques, elles sont généralement de découverte fortuite, lors d’un bilan d’évolution d’un cancer primitif connu [5], comme c’était le cas de notre patient chez qui le diagnostic de métastase splénique était de découverte fortuite lors de la surveillance radiologique. Sur le plan radiologique: les métastases ont le plus souvent un aspect hypo-échogène à l’échographie et hypo-dense au scanner, elles peuvent être uniques ou multiples, peuvent siéger soit dans le parenchyme, soit dans la capsule ou les deux à la fois. Le PET scan reste la technique la plus sensible surtout en cas de métastases non décelables aux autres techniques d’imagerie, ou en cas d’élévation isolée d’ACE sans lésions détectées par les autres moyens d’imagerie [5]. Le diagnostic des métastases spléniques est le plus souvent un diagnostic de présomption, certains auteurs ont proposé une ponction biopsie de la rate pour avoir un diagnostic histologique, mais pour la plupart, la confirmation diagnostique est basée sur la splénectomie, qui va permettre de traiter les complications et, peut-être, de contrôler l’évolution de la maladie cancéreuse [6], comme c’est le cas de notre observation où le diagnostic de métastase splénique n’a pu être confirmé que sur la pièce de la splénectomie.

Sur le plan thérapeutique: La splénectomie suivie d′une chimiothérapie systémique semble le traitement de choix des métastases spléniques isolées d′un carcinome colorectal [7] ce qui rejoint notre cas. Selon les données disponibles dans la littérature, les auteurs suggèrent une amélioration significative de la survie à long terme après la splénectomie pour les métastases spléniques métachrones d’un carcinome colique [8], mais on ne connait pas le taux de récidive après la splénectomie. Par contre le pronostic des métastases spléniques synchrones semble être lié au stade avancé de la maladie. Vue la rareté des cas rapportés dans la littérature, aucune conclusion ne peut être établi.

Le progrès des chimiothérapies et l’arrivée de thérapies ciblées ne doit pas faire oublier que seule la résection chirurgicale offre une possibilité de guérison. L’emploi d’une chimiothérapie péri opératoire peut se faire comme pour les métastases hépatiques et pulmonaires, et reste discutable en fonction de la résecabilité de la métastase [9]. Le choix du protocole est en fonction de la chimio sensibilité antérieure, de la toxicité cumulative et des suites opératoires [10].

## Conclusion

Les métastases spléniques, bien que rares, doivent être recherchées lors du suivi de tout cancer viscéral primitif. L’exploration de la rate devrait également être systématique lors de toute laparotomie. La splénectomie totale est un moyen efficace de faire le diagnostic définitif de ces métastases et de les traiter afin de prévenir les complications et d’améliorer la survie.
